# Neighborhood Disadvantage, Brain, and Neurocognitive Function: Relations Among Cognitively Normal Older Adults

**DOI:** 10.1093/geroni/igaa057.1922

**Published:** 2020-12-16

**Authors:** Regina Wright, Desiree Bygrave, Shari Waldstein, Christos Davatzikos, Guray Erus

**Affiliations:** 1 University of Delaware, Newark, Delaware, United States; 2 University of Maryland Baltimore County, Baltimore, Maryland, United States; 3 University of Pennsylvania, Philadelphia, Pennsylvania, United States

## Abstract

Neighborhood disadvantage has been linked to poor health; however, research has not adequately examined its influence, across multiple domains of disadvantage, on neurocognitive function and underlying brain health in older adults. Thus, the objective was to examine associations between neighborhood disadvantage, brain health, and neurocognitive performance, and examine age and sex as effect modifiers. The analysis included 136 older adults with a mean age of 68.04y who underwent neuropsychological and psychosocial testing and 3T cranial magnetic resonance imaging. Neighborhood disadvantage was characterized using the Area Deprivation Index (ADI). Multivariable regression analysis, adjusted for age, sex, education, and depression, showed that greater ADI score (greater disadvantage) was associated with poorer working memory performance (p<.05) and lower hippocampal volumes (p<.05), with no evidence of effect modification. Results suggest that greater neighborhood disadvantage may play a role in working memory and brain structure, which are vital to quality of life in older adulthood.

